# Impact of region-of-interest size and location on quantitative contrast-enhanced ultrasound of canine splenic perfusion

**DOI:** 10.1186/s12917-021-02973-z

**Published:** 2021-08-11

**Authors:** Simona Morabito, Simona Di Pietro, Luca Cicero, Annastella Falcone, Luigi Liotta, Rosalia Crupi, Giovanni Cassata, Francesco Macrì

**Affiliations:** 1 Department of Veterinary Sciences, University of Messina, Polo Universitario Annunziata, 98168 Messina, Italy; 2 Istituto Zooprofilattico Sperimentale della Sicilia “A. Mirri”, Via Gino Marinuzzi 3, 90100 Palermo, Italy

**Keywords:** Coefficient of variation, Perfusion-related parameters, Dog, Spleen, Sonovue, Qontrast

## Abstract

**Background:**

During contrast enhanced ultrasound (CEUS), the features of the regions of interest (ROI) can affect the value of the perfusion-related parameters obtained from a time intensity curve (TIC). In veterinary medicine, conflicting have been reported on the influence of ROI size and location on renal CEUS. There are some disagreeing evidences regarding the optimal method for selecting ROI in quantitative analysis of renal perfusion using CEUS.

The aim of this study was to evaluate the effect of the size and location of ROIs in the spleen of conscious dogs on perfusion variables determined using sulphur hexafluoride contrast-enhanced ultrasounds.

**Results:**

A prospective observational study on 15 client-owned mixed-breed adult dogs was performed using a system equipped with contrast-tuned imaging technology. Qualitative and quantitative assessments of the spleen enhancement pattern were carried out. Three square ROIs (0.05 cm^2^) were manually drawn in a row and spaced 1 mm apart, placing adjacent ROIs at three different depths. Three medium rectangular ROIs (0.3 cm^2^) include the 3 smallest ROIs in each row, indicated by the letters A, B and C, and a single large square ROI (1 cm^2^) was drawn containing all previous ROIs. Software analysis of time-intensity curves generated within each ROI allowed us to calculate the perfusion-related parameters: peak enhancement, time to peak, regional blood flow, mean transit time and regional blood volume.

The coefficient of variation for all blood-related parameters was always lower in the larger ROI than in the other smaller ROIs. ROI A and B, positioned proximally and medially, levels respectively, showed similar coefficients of variation to the largest ROI. The analysis of variance model exhibited a significant effect of location and size of the ROIs in the quantitative analysis of canine spleen perfusion, with a reduction of perfusion-related parameters in the distal ROI.

**Conclusions:**

The recommendation for a quantitative CEUS examination of a dog’s spleen is to analyze splenic perfusion by drawing a sufficiently large ROI proximal to the ultrasound beam on the splenic parenchyma. This may be of clinical relevance in the diagnosis of splenic diseases.

## Background

Contrast-enhanced ultrasound (CEUS) is an imaging technique useful to obtain a reliable quantification of tissue perfusion based on region of interest (ROI) analysis. It is performed by injecting into the bloodstream gas-filled microbubbles, stabilized by an outer shell; these microbubbles cause an enhancement of the microcirculation, providing the quali-quantitative assessment of perfusion changes in an organ or tissue in real-time [1]. Second generation ultrasound contrast agents do not diffuse in the extra-vascular space, hence reflecting only the tissue vasculature [2]. The change of brightness over time is a function of the contrast agent inflow and outflow in a selected ROI. Data obtained with this method need to be processed with a dedicated software that allows to analyze signals from the blood-pool agents without background noise. Brightness and its distribution inside a ROI are analyzed in order to describe typical enhancement patterns for a tissue or lesion compared to the adjacent tissue and to obtain the perfusion-related parameters calculated from time intensity curves (TICs) in a selected ROI [1].

The standardization of TIC analysis is crucial to gain a reliable quantitative CEUS evaluation and clinical decision-making.

Ex vivo and in vivo studies reported that size and location of the ROI can affect the value of perfusion-related parameters computed from the TIC [3–6]. The ROI is drawn and localized manually by the operator, the size and placement are therefore variable.

In veterinary medicine, opposing results have been reported on the influence of ROI size and localization during renal CEUS on experimental models [4, 5, 7] and anaesthetized or conscious dogs [8–10]. There are some disagreeing evidences regarding the optimal method for selecting ROI in quantitative analysis of renal perfusion using CEUS. Leinonen et al. (2011) found that there was a significant inverse association between the size of the ROI and the peak intensity [3]. Other authors recommended the use of the largest possible ROI to minimize the influence of renal perfusion heterogeneity [11, 12]. Conversely, drawing three smaller ROIs inside the large ROI, the heterogeneity of diffusion of the contrast medium in the area was eliminated, evidencing the option to use small or large ROIs during renal CEUS in dogs [10].

Vascular patterns of normal or pathological spleen in healthy dogs and cats have been described using the CEUS analysis and recent studies have been focused on canine normal splenic perfusion patterns and blood-pool phase peculiarities of splenic lesions, with the aim of improving the diagnostic procedures in discriminating a benign from a malignant tumour, and in monitoring therapy [13–21]. However, no guidelines have been reported for the ideal placement or size of the ROI when measuring perfusion by CEUS in canine spleen.

The goal of this study was to evaluate the effect of the ROI size and location on perfusion-related parameters in the spleen in not-sedated dogs, based on the hypothesis that the ROI variables would significantly affect the mean value of canine splenic perfusion-related parameters.

## Results

All dogs included in the study showed haematological parameters within reference range, while haematochemical results exhibited a mild increase in blood urea and creatinine values. On B-Mode examination, enrolled dogs showed a normal splenic tissue echo-pattern. No focal lesions were identified, and the splenic parenchyma presented a widely homogeneous echo pattern. Colour Doppler detected a normal vasculature and excluded the presence of ischemic vascular lesions or intravascular thrombi.

**Fig. 1:**
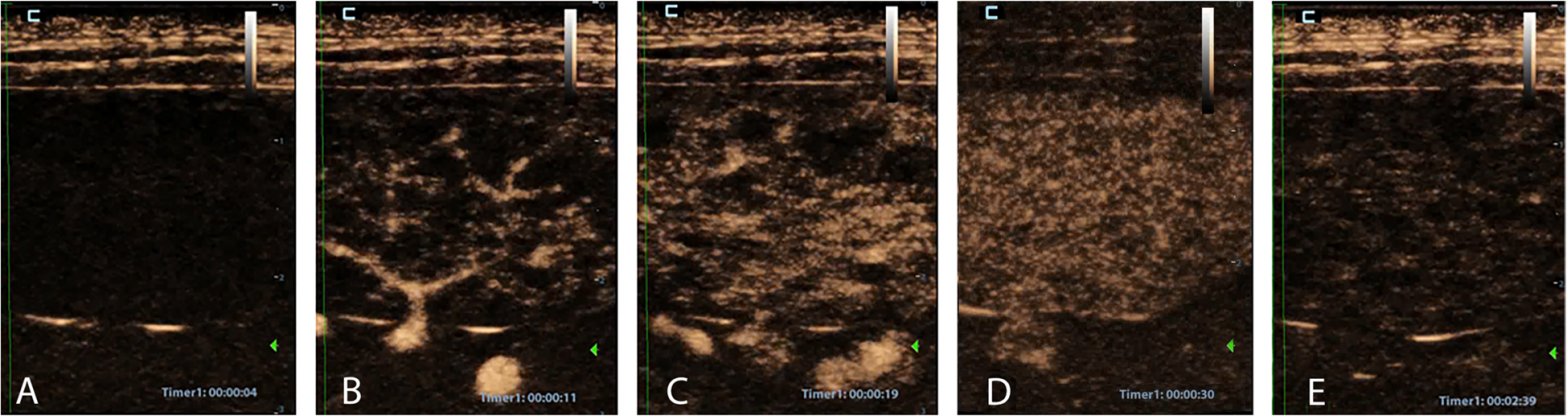
Representative images of the splenic vascularization pattern in a dog during the CEUS study. **A**: pre-contrast image after the bolus administration of contrast medium. **B**–**E**: post-contrast injection images over time

Contrast-enhanced ultrasound displayed a perfusion of the splenic tissue characterized by an early wash-in phase with a rapid enhancement of the small splenic arteries, 10 ± 2 s. (mean ± SD, standard deviation), a heterogeneous phase of enhancement of the spleen that became homogeneous at a mean peak enhancement of 45 ± 15 s. (SD), and a slow decrease of enhancement in the wash-out phase (Fig. 1). The absence of splenic lesions was confirmed by CEUS.

The post-processing quantitative analysis clarified the variations in signal intensity induced by the passage of the microbubbles and created parametric maps of the ROIs under investigation, through the signal processing of the wash-in and wash-out curves.

The coefficient of variation for all perfusion-related parameters was always lower in the largest ROI (ROI MAX) than in the other smaller ROIs. The coefficient of variation for peak enhancement in ROI MAX was 18 %, whereas for other perfusion-related parameters were equal or higher than 20 %.

The coefficient of variation for all blood perfusion-related parameters of ROI A and B, located at the proximal and median level, respectively, showed values similar to those of the ROI MAX, while the coefficient of variation for the distal ROI C parameters are generally higher than ROI MAX. Data are shown in Table 1.

**Table 1:** Coefficients of variation (%) for splenic perfusion-related parameters of the different ROIs

	Proximal ROI (A)	Middle ROI (B)	Distal ROI (C)	ROI Max
PEAK	19.46	18.43	21.58	17.89
TTP	24.36	25.59	30.51	22.12
RBV	36.49	38.67	41.68	36.94
RBF	23.92	23.66	24.16	20.76
MTT	29.23	24.48	25.35	21.68

*ROI* region of interest; *Peak* peak enhancement; *TTP* time to peak; *RBV* Regional blood volume; *RBF* Regional blood flow; *MTT* mean transit time

The mean values and standard deviation of each perfusion-related parameter in the examined ROIs, together with their statistical significance, are depicted in Table 2.

**Table 2:** Mean values (±SD) of each perfusion-related parameter in the largest ROI MAX and medium-size ROI A, ROI B, and ROI C with their statistical significance

Variable	ROI MAX	ROI A	ROI B	ROI C	*p*-value
Peak (%)	34.43±7.1^a^	35.31±6.8^a^	35.72±7.9^a^	31.89±6.9^b^	0.035
TTP (s)	45.88±11^a^	46.01±17^a^	46.88±11^a^	44.33±9.1^b^	0.032
RBF (L/min)	43.11±8.9^a^	45.67±11^a^	43.55±9.8^a^	41.10±8.8^b^	0.015
MTT (s)	76.09±17^a^	78.87±18^a^	78.35±16^a^	71.56±18^b^	0.021
RBV	3425.22±1309^a^	3545.88±1231^a^	3550.08±1311^a^	3073.87±1442^b^	0.003

Different letters (a–b) in the same row indicate significant differences by ANOVA test, followed by Tukey’s post-hoc test (*p* < 0.05)

The application of the analysis of variance (ANOVA) model exhibited a significant effect of the location and size of the ROIs in the quantitative analysis of canine spleen perfusion, with a significant decrease of mean values in ROI C compared to ROI A, ROI B, and ROI MAX.

Particularly, the comparison between ROI C and ROI A provided the following p-values: peak, *p* = 0.021; TTP = 0.022; MTT = 0.045; RBV = 0.042. Comparing ROI C with ROI B, the *p*-values ​​were the following: peak = 0.019; TTP, *p* = 0.044; MTT, *p* = 0.031; RBV, *p* = 0.019.

Furthermore, the p-values of the comparison between ROI C and ROI MAX were the following: peak = 0.024; TTP = 0.039; RBF = 0.014; MTT = 0.041; RBV = 0.011.

**Fig. 2:**
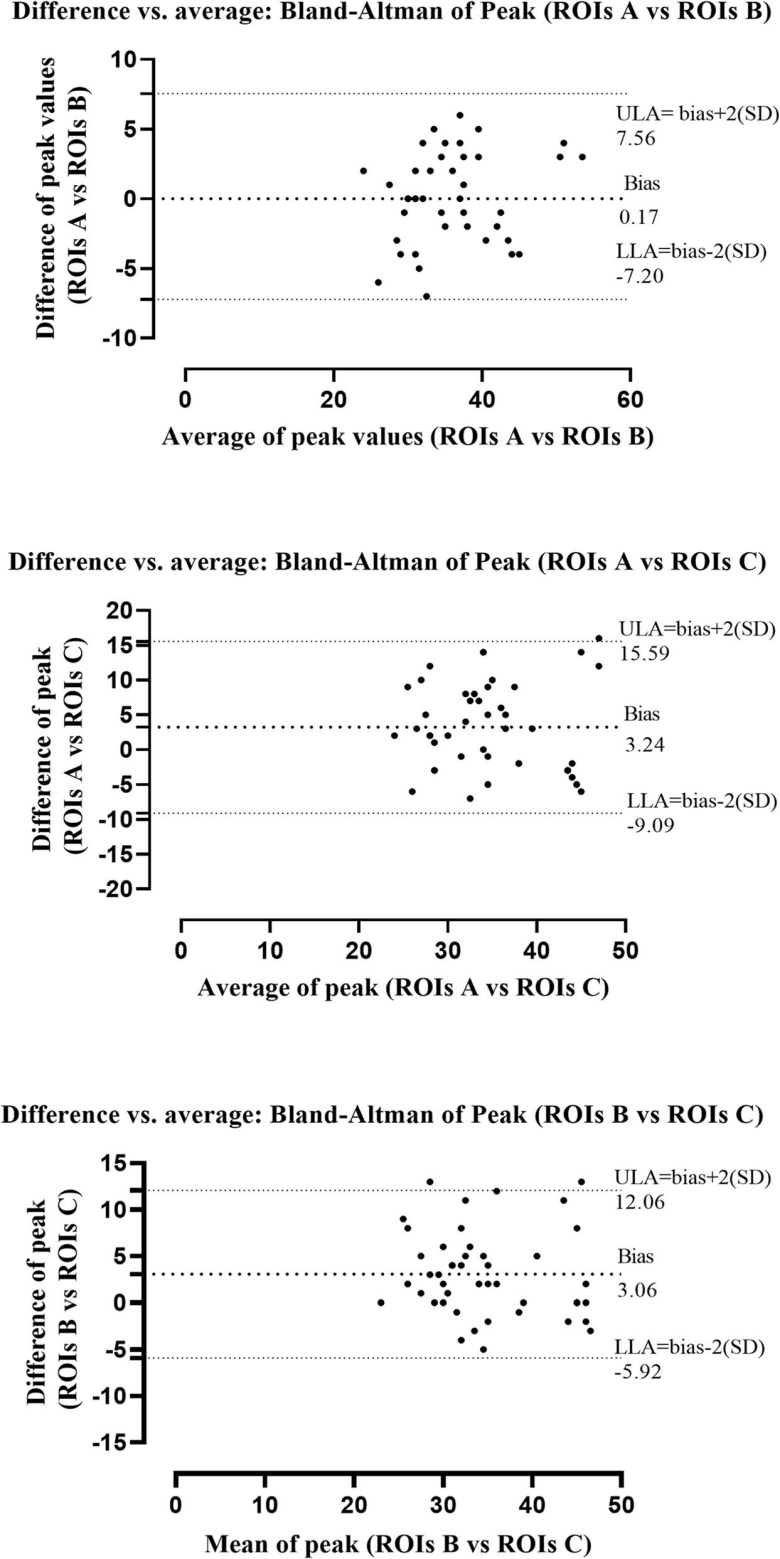
Graphical display of the Bland–Altman analysis of peak enhancement values CEUS-derived, comparing the ROIs at different depth of splenic parenchyma. Notes: The mean difference or the bias is the central dotted black line with large dots. The 95 % upper and lower limits of agreement are represented by the dotted black lines with small dots. Abbreviations: ULA, upper limit of agreement; LLA, lower limit of agreement

The Bland-Altman scatter plots displayed on a Cartesian diagram the relationship between the values of the differences of the ROI measurements at different depths (ordinate axis) and their mean (abscissa axis). The line relative to the mean of the differences of the ROI measurements (bias) and the lines corresponding to the limits of agreement of the bias (bias ± 2 SD) were also plotted. This test showed that measurements of all splenic perfusion-related parameters, visualized as points on the graph, were within the two lines of the confidence interval. For each perfusion-related parameter, the bias appears to change with the ROI depth, becoming higher as the depth increases. The Bland-Altman plots of peak enhancement values comparing perfusion-related variables in ROIs at different depths were reported in Fig. 2. The peak value (%) in the proximal ROI (ROI A) is, on average, 0.17 % greater than the intermediate ROI (ROI B) and 3.24 % greater than the distal ROI (ROI C). The standard deviation of the differences between the pairs of values measured in the ROIs at different depths was found to be 3.7 and the limits of agreement, calculated as 0.17 (bias) ± 2 × 3.7 (SD), ranged from − 7.20 to 7.56.

No adverse reactions during the procedure occurred.

## Discussion

In veterinary medicine, CEUS has become a reference method in imaging due to its remarkable safety in use, absence of ionizing radiation or toxicity and low costs [22].

Factors related to technical variables and contrast medium or patient-related factors are known to contribute to variability in quantitative analysis of CEUS and resulting perfusion-related parameters [20].

Technique-related factors (use of the three-way stopcock, catheter size, contrast medium injection rate, and volume of the saline flow) should be standardized to reduce their influence on time to peak values [9, 23]. The size of the organ and the characteristics of the parenchyma could also influence the acoustic “strength” of the beam and determine variations in the peak intensity [24].

In this study, procedures were standardized as much as possible, seeking to minimize the influence of bubble manipulation on quantitative imaging analysis.

According to previous studies, after injection of contrast medium, a saline solution was inoculated into the venous circulation in a standardized way regarding the use of stopcock, the consistency in the volume of the saline solution (a bolus of 5 mL) and the rapidity of its administration [9, 25].

All scanner parameters have been set: a low mechanical index has been chosen to minimize the disruption of the microbubbles and allow their accumulation in the microvasculature; this would also allow the same expert operators to perform the procedure every time, as suggested to avoid variability in clinical applications of CEUS [18, 20]. The time gain compensation and overall gain were decreased prior to injection of the contrast agent in order to suppress most of the background tissue signals.

Efforts have been made to minimize patient-related factors that can significantly influence peak enhancement and mean transit time. Dogs were enrolled in the study on the basis of ultrasound characteristics of the splenic parenchyma, which was homogenous in all subjects; moreover, the spleen is a superficial intra-abdominal organ, whose position is little influenced by the physical conformation of the animal.

Anaesthetic drugs can also modify the quantitative analysis of the contrast medium due to their pharmacokinetic and pulmonary metabolism, mainly when inhalers are used [15, 20]. Intravenous anaesthetics alter the CEUS quantitative analysis, altering blood pressure and heart rate: the peak intensity time appears to be much faster in awake than in dogs anesthetized with propofol [26]. In contrast, butorphanol did not affect cardiovascular parameters during the evaluation of feline renal perfusion with CEUS [15, 21, 27].

In our context, it is possible to exclude the effect on splenic blood flow of anesthetic or sedative drugs, as the dogs were awake.

In this study, the location and size of the ROI were analyzed in order to detect their effect on the variability of perfusion-related parameters. Our results showed that there was a deviation to measure values at different depths. If a ROI in a subjectively homogenous parenchyma area is shifted vertically, there are deviations for the resulting data, with a larger coefficient of variation at the deepest ROI. The deviations differ for different blood-related parameters in a range between 18 and 42 % with a mean deviation of 27 %. Peak enhancement appears to be the most stable parameter for depth positioning variations. This finding cannot be compared with previous data because there are no similar studies performed on the canine spleen in literature.

The lowest coefficient of variation for all perfusion-related parameters was found when a single large square ROI (1 cm^2^) was drawn on the splenic parenchyma, compared to the other smaller ROIs included in the previous one. Although there was still a tendency for some parameters such as peak enhancement to be more stable than others (coefficient of variation 17.89 %), all parameters had a coefficient of variation of less than 40 %, showing good stability against the influences of variations in size and depth. All blood-related parameters from the largest ROI showed acceptable values of over 60 % so that they can be considered more reliable, allowing for the detection of even small perfusion anomalies. The same was true for the perfusion-related parameters of the other ROIs except those drawn in the deepest level.

There are some practical clinical implications arising from these findings. The quantitative CEUS analysis of the canine splenic perfusion should not be performed in a deep level of parenchyma. Furthermore, it would be appropriate to draw a ROI as large as possible. When comparing more than one ROI, e.g. in a tumour vs. representative parenchyma they must be compared in the same depth. This is in accordance with previous CEUS studies for the positioning and size of ROI, performed in human liver and canine kidney (10, 29).

ANOVA analysis allowed us to better understand the observed variability of perfusion-related parameters: they were significantly greater in proximal ROIs than in distal ROIs. One reason for this finding is that the ultrasound beam may be attenuated in deeper areas [28].

Perfusion-related parameters appear to be independent from ROI size, if ROIs are positioned in proximal and middle levels of the splenic parenchyma.

The Bland-Altman test showed that all CEUS-derived quantitative parameters of canine splenic perfusion provided congruent results, particularly for peak enhancement. Comparing ROI A with ROI B, peak enhancement values were proximal to the 0 bias line with a small bias SD. The test showed that the mean difference between proximal and intermediate ROIs was lower than that between proximal and distal ROIs for all perfusion-related parameters examined, confirming the influence of depth on ROIs during quantitative evaluation. We believe that these differences may not be clinically acceptable during CEUS examination of the canine spleen.

Previous studies reported the influence of ROI management on CEUS-derived perfusion parameters of physiological and pathological tissues other than the spleen in both human and veterinary medicine [3, 5, 6, 10, 29–31]. Our results are the first data on the effect of ROI size and localization on parameters related to canine splenic perfusion, so that it is difficult to compare with different experimental settings and methods.

## Conclusions

The recommendation for a quantitative CEUS examination of a dog’s spleen is to analyze splenic perfusion by drawing a sufficiently large proximal to the ultrasound beam ROI on the splenic parenchyma. This may be of clinical importance in the diagnosis of splenic disorders.

When comparing more than one ROI, for example pathological versus representative parenchyma, they must be compared in the same depth. Perfusion-related parameters should not be analyzed in a depth.

Our results could be useful to define guidelines for the selection of ROIs and to control the variability in the use of CEUS to evaluate splenic perfusion.

## Methods

### General materials

The signed informed consent of the dog owners about methods and purposes of this study was obtained. The protocol was approved by the Animal Ethics Board of the Department of Veterinary Sciences, University of Messina (protocol number: 13/2017). All treatments, housing and animal care followed the EU Directive 2010/63/EU on the protection of animals used for scientific purposes.

Fifteen client-owned mixed-breed adult dogs were recruited prospectively for this study, and presented at the Veterinary Teaching Hospital of the University of Messina between October 2017 and February 2019 for assessment of renal perfusion by CEUS. Dogs were included in the study only if their spleen was homogenous at ultrasonography. Dogs with large focal or multifocal splenic lesions were excluded. Dogs that met the inclusion criteria were 8 males and 7 females, ranging in age from 1 to 7 years and a mean body weight of 30.9 ± 3.6 (SD) kg.

For this study, we used the sample size recommended in previous statistical studies [32], which considered a sample homogeneity of 15 patients to be appropriate for statistical analysis. In order to assess the health status of the dogs, a complete clinical examination and haemato-biochemical screening were performed on each animal enrolled. Heart and respiratory rates, blood pressure, and capillary filling time were recorded; laboratory tests included complete blood counts (CBC) and biochemical profile (urea, creatinine, total protein, albumin, glutamate pyruvate transferase (GPT), gamma glutamyl transferase (GGT), aspartate aminotransferase (AST).

### Ultrasonography procedure

All the dogs were subjected to B-mode ultrasonography, Doppler ultrasonography and CEUS. Ultrasound and Doppler examination were performed by the same investigator (FM) using a scanner Mindray M9 (Shenzhen, China), equipped with a linear probe (10-12-MHz). The dogs were not sedated and were manually restrained in right lateral recumbency, the hair was clipped, and alcohol (70 %) and coupling gel were applied to the skin.

Spleen tissue was considered normal if the margins were regular and smooth and the parenchyma showed a finely textured and homogeneous pattern, more echogenic than the liver and left kidney cortex. Colour Doppler was performed to evaluate splenic vascularization and rule out the presence of splenic intravascular thrombotic structures or ischemic lesions.

CEUS examination was carried out using a linear transducer probe (10-12-MHz) with contrast agent capability. The contrast agent, INN-sulphur hexafluoride (SonoVue®, Bracco International, Milan, Italy), was prepared following the manufacturer’s recommendations and was quickly injected (0.05 mL/kg body weight) via a three-way stopcock and an 18-gauge catheter placed in the cephalic vein, according to a previously reported methodology [33, 34]. Each dog received two bolus injections of contrast agent, which were standardized and administered by the same investigator (SD). The first bolus was administered to assess the kidney while the second bolus, injected approximately 5–10 min after the first bolus, was used for the assessment of the spleen. The contrast injection was immediately followed by a 5 mL saline flush, as previously described [10].

Injection of the contrast agent and activation of a timer were started simultaneously and video clips were recorded for 2 min. Since the animals were not sedated, care was taken to keep the probe in the same position for at least 2 min. The spleen was observed with a mechanical index set at a low value (0.09). A single focal zone was placed in the deepest part of the spleen. The overall gain and time-gain compensation have been set so that no signal is obtained from the underlying splenic parenchyma. To identify the splenic capsule as an anatomic reference, its background signal was maintained.

**Fig. 3:**
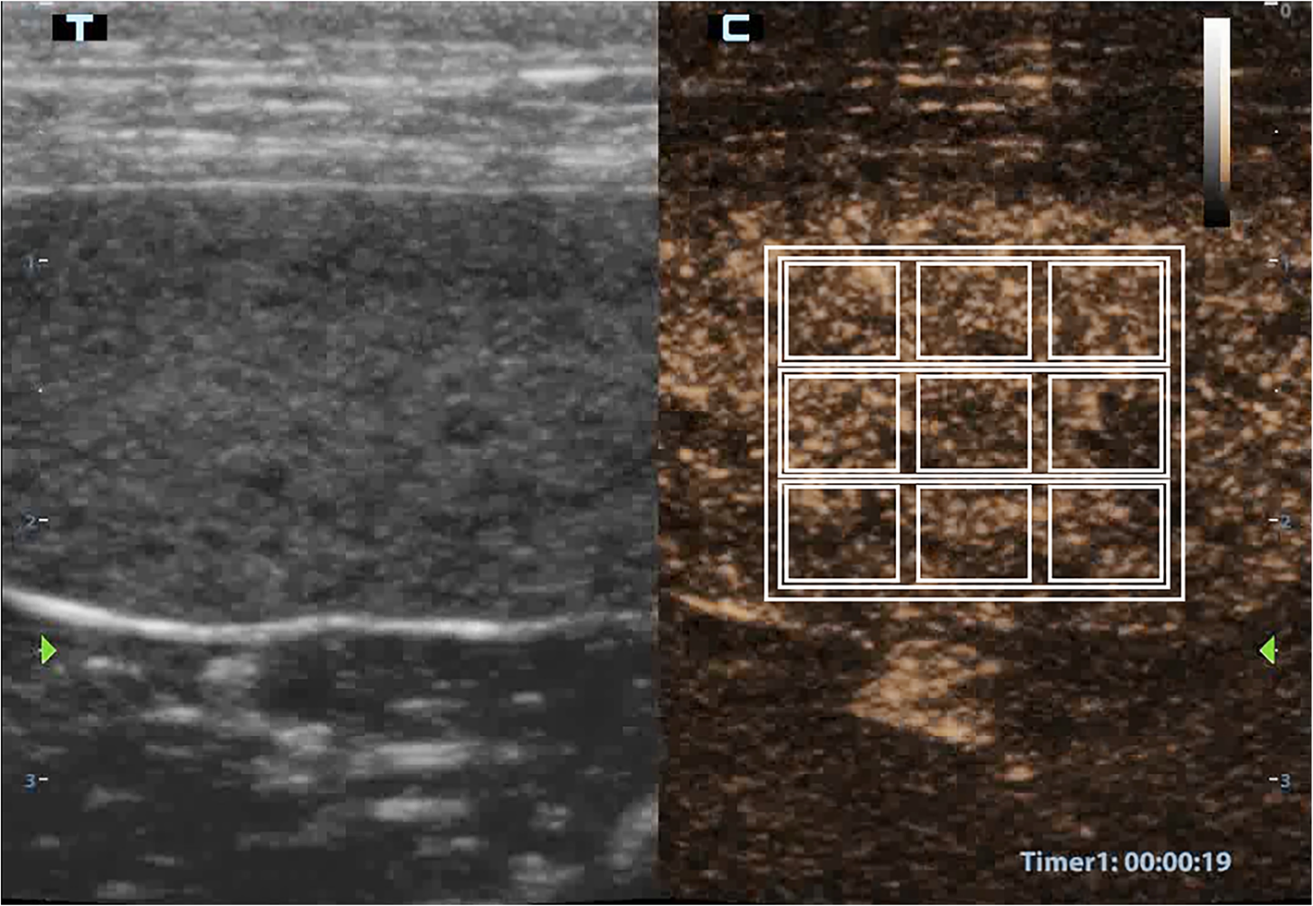
Representation of the ROIs used in the quantitative CEUS analysis of the canine splenic parenchyma. Nine square ROIs (1–9) each measuring 0.05 cm^2^ are separated by 0.1 cm. Three larger rectangular ROIs (A-C) have an area of 0.3 cm^2^, and each encloses a row of three smaller ROIs. A single large square ROI encompasses all others with an area of 1 cm^2^

Raw data (good quality video clips) obtained during the CEUS were digitally stored on a hard disk. A trained investigator (LL) analyzed all functional data. A qualified operator (SM) drew a total of 13 quadrangular or rectangular ROIs. The smaller ROIs (0.05 cm^2^) were numbered in sequential numerical order from 1 to 9. They were drawn in groups of three, at a distance of one millimetre, on three different depth levels: proximal, middle and distal. Around each group of three ROIs, a larger ROI (0.3 cm^2^) was drawn, indicated by the letters A, B and C. The total of 12 ROIs were grouped into a higher ROI (1 cm^2^), named ROI MAX (Fig. 3). The use of image analysis software resulted in post-processing analysis (Qontrast®, Bracco Imaging, Milan, Italy). This software processed the raw data enabling measurement of tissue perfusion in ROIs and automatic determination of variables. A time-intensity curve was also generated for each ROI, which is a parametric curve of time versus signal intensity (SI). The SI of a white band in the grey scale bar (8 bit) was defined as maximal (100 %). The software generated the following blood-related parameters: peak enhancement, time to peak (TTP), mean transit time (MTT), regional blood volume (RBV), and regional blood flow (RBF) for each ROI. These parameters were defined as follows:

- peak enhancement as the percentage increase in SI (from 0 to 100 as maximal intensity) reached during the transit of the contrast agent at a specific time point;
- TTP as the interval until the SI maximum of the contrast agent;
- MTT as the circulation time of the contrast agent in the examined tissue;
- RBV as the blood volume proportional to the area under the curve (AUC), defined as the area under the time intensity curve during the wash-in and wash-out phase;
- RBF as the ratio between RBV and MTT.

### Statistical analysis

Descriptive statistical analysis of the quantitative CEUS-derived parameters revealed a normal distribution for each value. Data obtained from the quantitative CEUS-derived parameters were subjected to statistical analysis, using XLSTAT PRO 5.7 (Addinsoft, New York, USA) and were subjected to ANOVA, which included the fixed effect of the ROIs (Max, A, B, C) and the random effect of the single dog. Mean values (± SD) of each perfusion-related parameter of the largest ROI MAX and medium-size ROIs (A, B, and C) were calculated. Comparisons between mean values were performed using Tukey’s post-hoc test. Statistical significance was set at p < 0.05. To define the influence of the location and size of the ROI on the perfusion-related parameters, the coefficient of variation (CV), expressed as a percentage, was calculated for each of them by applying the formula: CV = SD/mean x 100. The coefficient of variation ranged from 1 to 100 %; distributions with the lowest coefficient of variation were considered to have low variance. If a parameter showed the lowest coefficient of variation, it was considered acceptable and reliable.

Bland-Altman plots were performed by using software (Graphpad Prism 9.0.2) to carry out a discriminant analysis to highlight the mean differences between ROIs and depth.

## Data Availability

The datasets used and/or analysed during the current study are available from the corresponding author on reasonable request.

## Abbreviations

CEUS: Contrast-enhanced ultrasonography
MTT: Mean transit time
RBF: Regional blood flow
RBV: Regional blood volume
ROI: Region of interest
SI: Signal Intensity
TIC: Time-intensity curve
TTP: Time to peak

## References

[1] Haers H, Saunders JH (2009). Review of clinical characteristics and applications of contrast-enhanced ultrasonography in dogs. J Am Vet Med Assoc.

[2] Doury M, Dizeux A, de Cesare A, Lucidarme O, Pellot-Barakat C, Bridal SL, Frouin F (2017). Quantification of tumor perfusion using dynamic contrast-enhanced ultrasound: impact of mathematical modeling. Phys Med Biol.

[3] Leinonen MR, Raekallio MR, Vainio OM, Ruohoniemi MO, O’Brien RT (2011). The effect of the sample size and location on contrast ultrasound measurement of perfusion parameters. Vet Radiol Ultrasound.

[4] Claudon M, Barnewolt CE, Taylor GA, Dunning PS, Gobet R (1999). Ba- dawy AB. Renal blood flow in pigs: changes depicted with contrast-enhanced harmonic US imaging during acute urinary obstruction. Radiology.

[5] Taylor GA, Barnewolt CE, Claudon M, Dunning PS (1999). Depiction of renal perfusion defects with contrast-enhanced harmonic sonography in a porcine model. Am J Roentgenol.

[6] Mule S, De Cesare A, Lucidarme O, Frouin F, Herment A (2008). Regularized estimation of contrast agent attenuation to improve the imaging of microbubbles in small animal studies. Ultrasound Med Biol.

[7] Dong Y, Wang W, Cao J (2013). Quantitative evaluation of contrast-enhanced ultrasonography in the diagnosis of chronic ischemic renal disease in a dog model. PLoS ONE.

[8] Haers H, Smets P, Pey P (2011). Contrast harmonic ultrasound appearance of consecutive percutaneous renal biopsies in dogs. Vet Radiol Ultrasound.

[9] Waller KR, O’Brien RT, Zagzebski JA (2007). Quantitative contrast ultrasound analysis of renal perfusion in normal dogs. Vet Radiol Ultrasound.

[10] Macrì F, Di Pietro S, Liotta L (2016). Effects of size and location of regions of interest examined by use of contrast-enhanced ultrasonography on renal perfusion variables of dogs. Am J Vet Res.

[11] Wei K, Le E, Bin JP (2001). Quantification of renal blood flow with contrast- enhanced ultrasound. JACC.

[12] Schneider AG, Calzavacca P, Schelleman A (2014). Contrast-enhanced ultrasound evaluation of renal microcirculation in sheep. Intensive Care Med Exp.

[13] Ohlerth S, O’Brien RT (2007). Contrast ultrasound: general principles and veterinary clinical applications. Vet J.

[14] Nakamura K, Sasaki N, Yoshikawa M (2009). Quantitative contrast-enhanced ultrasonography of canine spleen. Vet Radiol Ultrasound.

[15] Rossi F, Fina C, Stock E (2016). Effect Of Sedation On Contrast-Enhanced Ultrasonography Of The Spleen In Healthy Dogs. Vet Radiol Ultrasound.

[16] Rossi F, Leone VF, Vignoli M (2008). Use of contrast-enhanced ultrasound for characterization of focal splenic lesions. Vet Radiol Ultrasound.

[17] Ohlerth S, Dennler M, Ruefli E (2008). Contrast harmonic imaging characterization of canine splenic lesions. J Vet Intern Med.

[18] Ivancic M, Long F, Seiler GS (2009). Contrast harmonic ultrasonography of splenic masses and associated liver nodules in dogs. J Am Vet Med Assoc.

[19] Mangano C, Macri F, Di Petro S (2019). Use of contrast-enhanced ultrasound for assessment of nodular lymphoid hyperplasia (NLH) in canine spleen. BMC Vet Res.

[20] Tang MX, Mulvana H, Gauthier T (2011). Quantitative contrast-enhanced ultrasound imaging: a review of sources of variability. Interface Focus.

[21] Leinonen MR, Raekallio MR, Vainio OM (2011). Effect of anesthesia on contrast-enhanced ultrasound of the feline spleen. Vet J.

[22] Seiler GS, Brown JC, Reetz JA (2013). Safety of contrast-enhanced ultrasonography in dogs and cats: 488 cases (2002–2011). J Am Vet Med Assoc.

[23] Talu E, Powell RL, Longo ML (2008). Needle size and injection rate impact microbubble contrast agent population. Ultrasound Med.

[24] Bargellini P, Orlandi R, Paloni C (2013). Contrast-enhanced ultrasonographic characteristics of adrenal glands in dogs with pituitary-dependent hyperadrenocorticism. Vet Radiol Ultrasound.

[25] Hwang J, Kang K, Kang J, Nam J, Park S, Yoon J, Choi M (2019). Effect of catheter diameter and injection rate of flush solution on renal contrast-enhanced ultrasonography with perfluorobutane in dogs. Am J Vet Res.

[26] Nyman HT, Kristensen AT, Kjelgaard-Hansen M (2005). Contrast-enhanced ultrasonography in normal canine liver. Evaluation of imaging and safety parameters, Vet Radiol Ultrasound.

[27] Stock E, Vanderperren K, Van der Vekens E, Haers H, Duchateau L, Polis I, Hesta M, Saunders JH (2014). The effect of anesthesia with propofol and sedation with butorphanol on quantitative contrast-enhanced ultrasonography of the healthy feline kidney. Vet J.

[28] Aldrich JE (2007). Basic physics of ultrasound imaging. Crit Care Med.

[29] Ignee A, Jedrejczyk M, Schuessler G, Jakubowski W, Dietrich CF (2010). Quantitative contrast enhanced ultrasound of the liver for time intensity curves-Reliability and potential sources of errors. Eur J Rad.

[30] Leng X, Huang G, Ma F, Yao L (2017). Regional Contrast-Enhanced Ultrasonography (CEUS) Characteristics of Breast Cancer and Correlation with Microvessel Density (MVD). Med Sci Monit.

[31] Lamby P, Jung F, Graf S, Schellenberg L, Falter J, Platz-da-Silva N, Schreml S, Prantl L, Franke RP, Jung EM (2017). Effect of iodinated contrast media on renal perfusion: A randomized comparison study in pigs using quantitative contrast-enhanced ultrasound (CEUS). Sci Rep.

[32] John E (2003). Sample size estimation: How many individuals should be studied?. Radiology.

[33] Quartuccio M, Mangano C, Macrì F (2018). Contrast-enhanced ultrasound evaluation of testicular interstitial cell tumours in conscious non-sedated dogs. Vet Med.

[34] Macrì F, Di Pietro S, Mangano C (2018). Quantitative evaluation of canine urinary bladder transitional cell carcinoma using contrast-enhanced ultrasonography. BMC Vet Res.

